# Conductive Cellulose based Foam Formed 3D Shapes—From Innovation to Designed Prototype

**DOI:** 10.3390/ma12030430

**Published:** 2019-01-31

**Authors:** Sanna Siljander, Pasi Keinänen, Anastasia Ivanova, Jani Lehmonen, Sampo Tuukkanen, Mikko Kanerva, Tomas Björkqvist

**Affiliations:** 1 Faculty of Engineering and Natural Sciences, Tampere University, P.O. Box 589, 33101 Tampere, Finland; pasi.keinanen@tuni.fi (P.K.); mikko.kanerva@tuni.fi (M.K.); tomas.bjorkqvist@tuni.fi (T.B.); 2 Department of Design, Aalto University, P.O. Box 31000, 00076 Aalto, Finland; anastasia.ivanova@aalto.fi; 3 VTT Technical Research Centre of Finland Ltd, P.O. Box 1000, 02044 VTT, Finland; jani.lehmonen@vtt.fi; 4 Faculty of Medicine and Life Sciences, Tampere University; P.O. Box 589, 33101 Tampere, Finland; sampo.tuukkanen@tut.fi

**Keywords:** nanocellulose, carbon nanotube, foam forming, conductivity, Salmiakki

## Abstract

In this article, we introduce for the first time, a method to manufacture cellulose based electrically conductive non-woven three-dimensional (3D) structures using the foam forming technology. The manufacturing is carried out using a minimum amount of processing steps, materials, and hazardous chemicals. The optimized solution applies a single surfactant type and a single predefined portion for the two main processing steps: (1) the dispersing of nanocellulose (NC) and carbon nanotubes (CNT) and (2) the foam forming process. The final material system has a concentration of the used surfactant that is not only sufficient to form a stable and homogeneous nanoparticle dispersion, but it also results in stable foam in foam forming. In this way, the advantages of the foam forming process can be maximized for this application. The cellulose based composite material has a highly even distribution of CNTs over the NC network, resulting a conductivity level of 7.7 S/m, which increased to the value 8.0 S/m after surfactant removal by acetone washing. Also, the applicability and a design product case ‘Salmiakki’ were studied where the advantages of the material system were validated for a heating element application.

## 1. Introduction

Scientific and industrial research communities are giving considerable attention to smart and functional materials that are based on renewable bio-based resources and are processed in eco-friendly ways. Cellulose is a very potential substance as it is a bio-based material and it can be used as a matrix when manufacturing functional structures. In addition, scientific and technological development is mature for the emergence of controlled multi-length materials that take advantage of different forms of cellulose applied in optimal ways. The added functionality requires developing new manufacturing processes and, especially, adjusting the colloid dispersion upon material production [1,2]. Our innovation is to use nanocellulose (NC) as a carrier for carbon nanotubes (CNT) in the foam forming process. In this article, we introduce for the first time a method to manufacture cellulose based non-woven three-dimensional (3D) structures that are electrically conductive. The manufacturing of the conductive non-wovens is done using a minimum amount of processing steps, materials, and hazardous chemicals. Our target is to use the selected surfactant type and amount in both the dispersion processing step and in the foam forming step. The concentration of surfactant must be sufficient to form stable and homogeneous dispersion, but it should also allow the formation of stable foam. 

The foam forming process has been studied since it was invented in the 1970s [3,4]. VTT Technical Research Centre of Finland has been active in the upscaling of foam forming technology and the first dynamic foam forming studies of the upscaling process were carried out with a modified semi-pilot scale former [5]. The foam forming technology enables the production and combination of a vast variety of fibre-based materials and the application of moulding technologies to form lightweight cellulosic 3D structures. In general, the foam forming technology utilizes aqueous foam instead of water as a carrier medium and the shear thinning behaviour of the foam makes it an excellent transport medium for fibres and particles, and it enables the excellent formation of the product being produced [6,7]. In addition, the air content of the carrier foam is 60–70% and it consists of air bubbles with a diameter below 100 µm. As stated in an article [8], interest is growing in the use of aqueous foam as a transporting medium of furnishes. By using the foam forming technology, a decrease in the cost of production and material savings can be achieved [6]. Furthermore, foam forming ensures structures that have excellent formation and, when combined with moulding technology, enables lightweight structures [9,10,11] and completely new functional product opportunities. 

The functional cellulose based matrix structure can be done by adding organic or inorganic nanostructured components. As one of the numerous options, carbon nanotubes (CNT) provide excellent electrical properties. CNTs have potential applications in electronics, for example, as interconnects [12] or energy storages [13]. The true conductivity potential of CNTs can be uncovered when the dispersing step to a liquid medium is performed properly to form a percolation network. Recent studies have revealed that the process of dispersing CNTs in a water-based system can be optimised so that the outcome functionality increases exponentially [14,15]. It is reported in those articles that, when using sonication as a dispersing process, the applied sonication energy to the dispersion and a properly selected surfactant play a key role when optimizing conductivity properties and dispersion quality. In the event, where cellulose pulp and NC-CNT dispersion are combined in the foam forming process, it is crucial to have the right concentration of surfactant to ensure there is enough air in the foam. Thus, a highly homogeneous dispersion of pulp fibres must be reached. The selection of the surfactant must be based on a systematic study to ensure that it will function as a dispersing aid in the sonication process but also form stable foam upon foam forming. 

One of the most commonly used surfactants for foam forming is the anionic surfactant sodium dodecyl sulphate (SDS), which is used in many industrial applications, such as shampoos, toothpastes, and shaving creams. The motive for the wide use is its relatively low price, foam stability, easy diffusion in water and therefore it can be used in rapid foaming [16]. However, SDS might still not be the optimal surfactant, since it has been shown that SDS as a surfactant of a water-CNT system does not disperse CNTs optimally to homogeneous dispersion, even if the higher sonication energy and concentration of surfactant are used [15]. The results in the cited study also clarify that, when surfactant Triton X-100 is used, stable homogeneous CNT-water dispersion can be achieved. 

When the target is to form stable and homogeneous dispersion of CNTs and nano-fibrillated cellulose, basically two alternatives of non-ionic surfactants exist, in addition to the basic case where nanocellulose itself acts as a dispersing agent [17]. Based on the current literature [14,18], Pluronic F-127 and Triton X-100 surfactants have resulted in good conductivity values when NC-CNT dispersion is used to manufacture nanocomposite films with relatively low CNT concentration. Actually, the conductivity values of the nanocomposite film can be further increased by removing the surfactant e.g., by acetone washing. This effect was seen in samples when NC and CNTs were dispersed while using surfactant Triton X-100, but not in samples that were dispersed using Pluronic F-127. The reason for this might be that Pluronic F-127 surfactant prefers nanocellulose over carbon nanotubes and, respectively, Triton X-100 prefers carbon nanotubes. Removing Triton X-100 surfactant from the interfaces improves the conductivity values.

Nanocellulose interactions with different surfactant types are reported comprehensively in the review article [19]. In general, interaction between nanocellulose and surfactant has a dependency on nanocellulose isolation procedure, which affects the surface charge and crystalline level of cellulose structure, which results as mobility in the wet state. Surface modification is needed to achieve interaction between different phases but often this is done at the cost of a given environmental effect, which is reached using nanocellulose as a structural component in composite structures. Using nanocellulose in papermaking processes has several advanced properties: it has a high aspect ratio, high strength along with good flexibility, an interaction potential via hydrogen bonding, and a tendency to form strong entangled networks. These properties mean that a high tensile strength is attainable at relatively low concentrations, lower than 5% with respect to pulp content [20,21]. Moreover, an increase in density has also been reported when nanocellulose is added to pulp. Nanocellulose attaches to the fibre surfaces as a layer and in this way a large contact area is formed, which increases the number of hydrogen bonds [22]. Using nanocellulose as a carrier for another material is mentioned in the patent filed by Tokushu Paper Manufacturing Co Ltd. They suggested using nanocellulose in tinted papers as a carrier for a dye or pigment [23]. Research group Hii et al. have reported in their article that microfibrillated cellulose contributed to the bonding of calcium carbonate filler in the fibre network of paper [24]. Nanocellulose is also suggested to be used as a biocarrier for controlled drug delivery [25]. 

The application of NC-CNT dispersions in the foam forming process enables the manufacturing of conductive 3D structures to almost any shape and size. Unfortunately, because of the nanoscale size of the CNTs, the formation with cellulose pulp alone does not occur efficiently enough to form the conductive percolation network during the foam forming process. When dispersion is prepared efficiently to form homogeneous dispersion without any aggregates present, the nanosized CNT particles flow through the cellulose fibre network in the vacuum assisted moulding with the foam. Our hypothesis is that nanocellulose can be used as a carrier for carbon nanotubes. It has been reported that nanocellulose can increase the strength of non-wovens that are manufactured using the foam forming process [6] and CNTs are good candidate to replace copper and aluminium as an interconnect material in the next generation electric devices [26].

When conductivity is reached to a certain level and an electric current is passaged through the structure, which acts like a resistive conductor, the system starts to heat due to its resistivity. This phenomenon is called resistive or Joule heating. Commonly personal heating elements are manufactured by inserting copper wire inside a seat heater or heating blanket. These types of multi-material structures are difficult to recycle, and heating occurs only near the conductive material. By harnessing carbon nanotubes as a conductive material for heating elements, it is possible to manufacture structures that do not create hot spots, act as a fire retardant, and the entire volume of the structure is functional (heating). Furthermore, it is possible to customize a maximum heating temperature of the heating element by adjusting the NC-CNT dispersion quality and the amount of CNTs in the foam formed structure.

## 2. Materials and Methods

In this study, nanocellulose production was based on the mechanical disintegration of bleached hardwood kraft pulp (BHKP). First, dried commercial BHKP produced from birch was soaked in water at approximately 1.7 wt % concentration and dispersed using a high shear Ystral dissolver for 10 min at 700 rpm. The chemical pulp suspension was prerefined in Masuko grinder (Supermasscolloider MKZA10-15J, Masuko Sangyo Co., Kawaguchi, Japan) at 1500 rpm and then fluidized with eight passes through Microfluidizer (Microfluidics M-7115-30 Microfluidics Corp., Westwood, MA, USA) using 1800 MPa pressure. The final material appearance of NC was a viscous and opaque gel. 

Multiwall carbon nanotubes were purchased from Nanocyl Inc. (MWCNT, Nanocyl 7000, Nanocyl SA., Sambreville, Belgium). CNTs were used as received, with-out pre-processing steps. This type of nanotubes is produced via catalytic chemical vapor deposition (CCVD). 

The selection of used surfactant in this study was done based on the previous studies [14,15] and the requirement to reach an efficient foaming capability. Therefore, Triton X-100 was selected and it is a non-ionic surfactant that has a hydrophilic polyethylene oxide chain and an aromatic hydrophobic group in its molecular structure. Triton X-100 was purchased from Sigma-Aldrich (Merck KGaA, Darmstadt, Germany). 

The NC and CNT were sonicated simultaneously and after sonication no centrifuge was used so that the preparation of aqueous dispersions could be achieved using a minimum amount of processing steps. Two identical sets of NC-CNT aqueous dispersions with a total volume of 1800 mL were prepared. One set contained NC (0.15%), CNTs (0.3%), deionized water, and surfactant Triton X-100, 0.4% (Table 1). The total dry mass for the dispersions was 8.25 g. The sonication of the dispersions was performed using a tip horn (ø 12.7 mm) sonicator Q700 with 20 kHz frequency (QSonica LLC., Newton, CT, USA) in 2000 mL glass beakers. The sonication amplitude of vibration (50%) was kept constant. The power output remained between 50 and 60 W for both sonications. The system included a water bath to keep dispersion cool during the sonication so that temperature would not rise above 30 °C. The water bath was cooled by circulating cooling glycerol through a chiller (PerkinElmer C6 Chiller, PerkinElmer Inc., Waltham, MA, USA). Dispersions were sonicated with energy per dry mass, respectively 700 kJ/g dry mass. The energy/dry mass indicates the total applied energy, not absorbed energy by dry mass. Part of the energy is used to heat the water and part is used to disperse nanoparticles. Also, part of the energy is used to degrade the sonicator itself and the vessel, to cause defects to the nanoparticles, and some energy is used to cause several different sonochemical reactions, like disintegration and the reorganization of water molecules [15,27].

The cellulosic fiber material used in this study was gently refined bleached kraft pulp (Scots pine 3.7% dry mass), as obtained from a Finnish pulp mill. 

**Figure 1:**
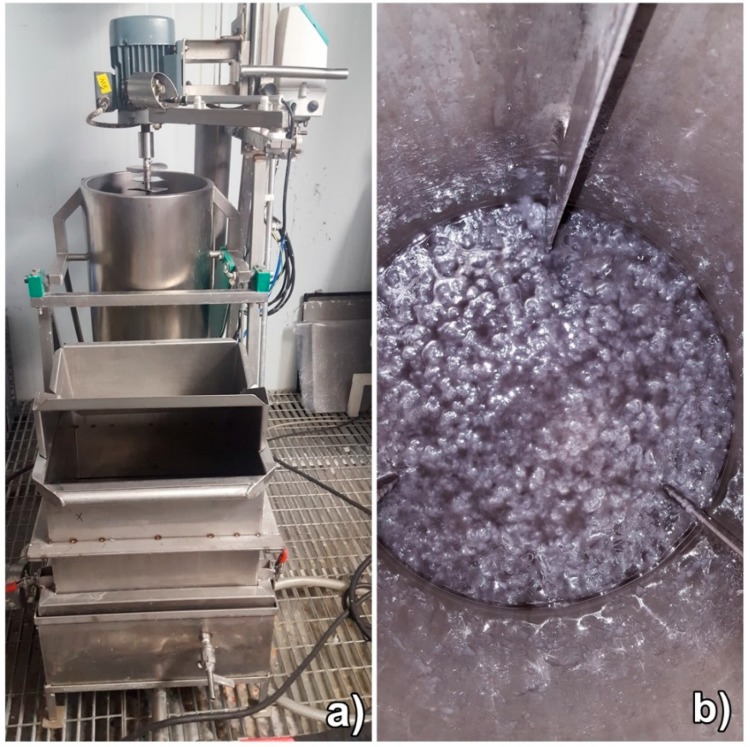
(**a**) Laboratory scale foam forming equipment; (**b**) nanocellulose-carbon nanotubes (NC-CNT) dispersion, pulp, and water mixture before foaming.

In the foam forming process, two sets of NC-CNT dispersion each volume of 1800 mL were poured in to the foam forming 32 cm diameter cylindrical tank (Figure 1a), followed by water and pulp, so that total volume was 5.5 litres (Figure 1b). Concentrations of materials are listed in Table 1. Mechanical mixing was carried out at a rotation speed of 3500 rpm for 3.5 min. Foaming time resulted in 70% air content to the foam, meaning that the total foam volume was 19 l. The prepared foam containing NC-CNT and pulp was poured into a planar mould that has a perforated surface with area of 0.19 m^2^, so that sheet formed 3D structure has a grammage of 100 g/m^2^. Wet foam was removed using 0.5 bar vacuum suction and for maintaining constant local suction plastic film was placed on the top of the foam column. After vacuum assisted foam removal the 3D cellulosic fiber sheet was dried in a thermal cabinet at 70 °C for 12 h until dry. Also, reference sheets using bleached kraft pulp and Triton X-100 surfactant were processed using the same procedure. 

The electrical conductivity of the foam formed non-wovens were measured using the four-probe measuring technique. With this method, it is possible to neglect the effect of contact resistances and thus provide more accurate conductivity measurements than using two-terminal measurement. The sheet resistances of prepared and cut foam formed non-wovens (size 30 mm × 30 mm) were measured using a four-point probe setup made in-house and a multimeter (Keithley 2002, Tektronix, Inc., Beaverton, OR, USA) in four-wire mode. The probes were placed in line, with equal 3 mm spacing. The four-probe setup is described elsewhere in detail [28]. The conductivity measurements were carried out using a 1 mA current and voltage was measured. Measurements were taken before and after the remaining surfactant was removed from samples by washing them in appropriate amount of acetone in room temperature (RT).

The mechanical testing (Testometric M500-25kN, Testometric Co Ltd, Rochdale, UK) of foam formed samples was done according to the standard EN 29073-3:1992 “Textiles. Test methods for non-wovens, Part 3: Determination of tensile strength and elongation”. From the foam formed non-wovens, ten sample pieces in total were cut (50 mm × 250 mm). Five of them were tested as such, while another set of five samples was washed in an appropriate amount of acetone in RT, so that the remaining surfactant Triton X-100 was removed. In general, the removal of surfactants from various nanocomposites can enhance the mechanical and electrical properties [14,29]. All of the non-woven samples were conditioned according to the standard ISO 139 before the tensile testing. The testing was performed by applying a constant rate of extension of 100 mm/min.

## 3. Results and Discussion

**Figure 2:**
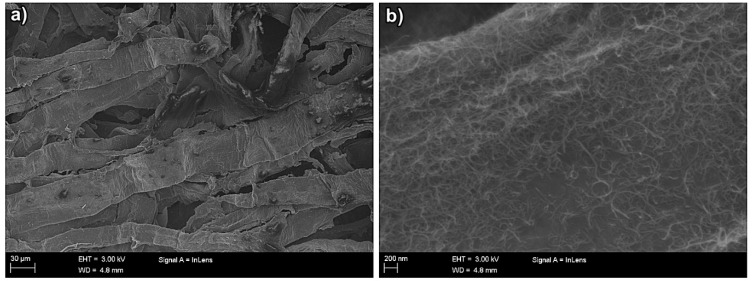
(**a**) Homogenous CNT coverage over cellulose fibers and (**b**) carbon nanotube percolation network on the surface of cellulose fiber.

SEM imaging (FE-SEM, 3 kW, Zeiss ULTRAplus, Oberkochen, Germany) was used to investigate the CNT distribution in the foam formed non-wovens. The SEM images have been taken from a dry sample and the NC has generated a network where the dimensions for individual fibers are very difficult to determine. Two different magnifications in Figure 2 are illustrating the NC and the CNT interaction, and the formation of a homogeneous CNT coverage over cellulose fibers. Figure 2b describes a type of CNT coating on NC: the coverage has excellent distribution over the surface. This means that the dispersion process using the specific sonication parameters has been successful and a conductive percolation network of CNTs is formed.

The electrical conductivity and tensile testing of the foam formed non-woven material was measured before and after the removal of the surfactant. The non-woven material containing surfactant was determined to possess a conductivity of 7.7 S/m (± 1.32). After the removal of the surfactant, conductivity increases to a value of 8.0 S/m (± 1.34). The mechanical strength of the formed non-woven material is of essential importance for any practical application. Here, the tensile strength of the foam formed non-woven was 121 N (± 11.8). After surfactant was removed, the strength increased to a value of 142 N (± 6.1). In comparison, the tensile strength for foam formed reference non-woven made using the wood pulp, without nanocellulose or carbon nanotubes, is 9.4 N (± 0.5), and after acetone washing, to remove the surfactant, the value increased to 16.5 (± 1.7). This shows that nanocellulose and carbon nanotubes also increase the tensile strength of the non-woven composite structure. Also, the mechanical strength was expected to increase after removal of the surfactant, due to the increased entanglement and interactions of nanocellulose and carbon nanotubes.

The effect of surfactant removal by acetone washing is minimal to mechanical strength and conductivity values. The reason for this might be that a majority of the surfactant is already removed from the structure during the vacuum assisted moulding process. Results also show that adding NC-CNT dispersion in the foam forming procedure with wood pulp will increase the tensile values of the manufactured non-wovens. This three material system has over ten times higher tensile strength compared to one material pulp system when residue surfactant is still present in the non-woven structure. 

The conductivity and mechanical performance of the final parameter set for the preparation led to results encouraging to the actual application verification. The high conductivity as well as mechanical strength mean that it is possible to manufacture conductive non-woven and utilize the innovated material system by using only two processing steps: (1) the sonication of the NC-CNT dispersion and (2) the foam forming of the final non-woven 3D structure.

## 4. Verification and Validation of Applicability: Heating Element “Salmiakki”

The verification study was carried out to manufacture a conductive 3D structure of a selected prototype using foam forming. The collaboration between designer and materials scientists has undergone the following steps:

1. Design briefing and setting the objective: a heating element
2. Research: material properties, benchmarking
3. Product innovation: user profiling (business case), user experience scenarios, product ideas, initial sketching, evaluation discussion, decision on the product features—heating element for indoor use and a device of a portable size and modular structure
4. Product design development: further sketching and paper mock-ups, evaluation discussion, decision on the visual form, clarifying the material specifications, testing of the material properties needed for the prototype manufacturing, CAD modelling, mold development for the foam forming process
5. Manufacturing and testing the prototype
6. Realistic rendering images of CAD model in interior settings
7. Establishing the outcomes of the collaborative process

Salmiakki, the heating element design taking advantage of conductive cellulose-based composite, is a result of close collaboration between a designer (A. Ivanova) and materials research scientists (S. Siljander and J. Lehmonen). The funding and targets of the product were a subtask in the national Design Driven Value Chains in the World of Cellulose (DWoC 2.0) project (www.cellulosefromfinland.fi). The objective was set to define the design of an electrical current-based heating element from newly developed conductive material that highlights the advantageous properties of this novel material and its production, provides a clear understanding of its future application possibilities, and emphasizes the local origin of raw materials that are used in the composite production. While features, such as high conductivity, no metal wires inside, temperature adjustability, mouldability, fire safety, light weight, soft material feeling, and recyclability make this material suitable for a variety of applications, several limiting factors, including restrictions in shape and color variations, needed to be considered to aim for the best performance and outlook.

Prior to the product ideation, a design research was conducted, which included benchmarking of existing indoor heating solutions, electrically-powered solutions, and solutions incorporating biomaterials. Benchmarking showed that a diverse variety of electrical interior heating designs are available on the market, however, biomaterials are seemingly not used for this type of applications. Based on a survey, no other product on the marked offered bio-based, biodegradable indoor heating product solution with flexible temperature adjustment, and fabric-like tactile properties. 

Product innovation was launched via creation of different user profiles. No in-depth user study was conducted prior to profile creation; the aim of profiles “personas”, was to serve as inspiration in the innovation and sketching the design process whilst helping to envision environments and situations where potential product’s users would benefit from the properties of conductive cellulose-based composite material. For instance, in addition to heating, such device could be used to keep places dry, serve as an acoustic element or room divider, and have a decorative purpose. As well as in home interiors, it could be used inside boats, cars, campervans, or other spaces that are protected from direct contact with water. Consequently, the decision was made to design a compact heating element of portable size and modular format, for indoor usage.

Visual design was initially carried out through the creation of sketches and scaled mock-ups, followed by full-scale paper models, which were discussed at the evaluation phase. Each paper model was considered for its functional and perceptional attributes, resulting in the choice of a diamond-like shape that has got the name “Salmiakki” due to its resemblance in shape and black color to a traditional Finnish candy.

For Salmiakki to be developed into a functional prototype, technical configurations of the material, direction of the electric current, and additional functional elements of the future heating device were outlined and observed when a CAD model was drawn. The CAD model was created for two major purposes: (1) to prepare a digital file for CNC-machinery of the mould for foam-forming process and (2) to create realistic 3D drawings to visualize the concept and plan modular compositions. 

In order to manufacture the prototype a mould for foam forming process was sized and prepared. As a reference, the process of mould design for foam-formed cellulose fiber materials was considered, as described by Härkäsalmi et al. To ensure successful formability during the foam-forming process, the mould must have a sturdy structure withstanding the vacuum pressure (suction), and correct permeability allowing for water to pass through while capturing the cellulose fibers [8]. To achieve this, the structure was designed to consist of two parts, both being female moulds: load-bearing supporting structure machined from polystyrene foam and a smooth surface layer with micro-perforation, vacuum-formed from a polypropylene sheet with a 1.5 mm thickness. The prototype was manufactured at pilot plan of VTT Technical Research Centre of Finland located in the City of Jyväskylä. 

**Figure 3:**
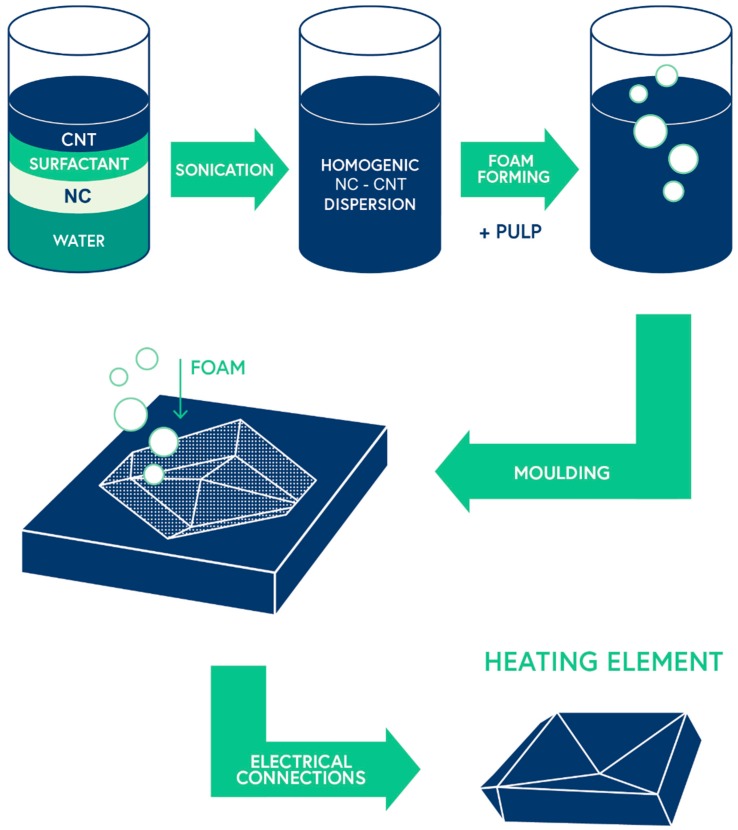
Manufacturing process of heating element prototype Salmiakki.

Material concentrations and methods used were the same as in foam forming 3D sheets, only pulp concentration was increased to 85 g dry mass, meaning a concentration of 1.5% in foam forming. In Figure 3, a schematic flow of manufacturing of heating element Salmiakki is presented.

The sheet resistivity of the manufactured 3D shape was measured using the four-probe method before and after acetone washing. Average sheet resistances with standard deviation were, when measurements were performed from eight sample locations, 26.1 Ω/sq (±2.7), and after acetone washing, 25.8 Ω/sq (±2.0). Acetone washing did not decrease resistivity much, meaning that there is minimum amount of surfactant present in the structure that does not alter the conductivity of the carbon nanotubes. A post treatment option was studied to enhance the visual features and the conductivity. The final 3D structure was coated by the spray of NC-CNT dispersion that resulted darker, black finish and alongside, enhanced heating properties at a lower electrical voltage. If all electric energy *P* is converted into heat, then heater temperature can be evaluated using Joule’s first law of heating P = U^2^ × R^−1^, where U (electric voltage input, V) and R (resistance, Ω). Using the electric voltage input of 18.5 V with the resistivity of 25.8 Ω/sq, electric power of heating element is 13.2 W. After the post treatment spray layer of NC-CNT dispersion, the resistivity decreased to value 11.2 Ω/sq (±0.9), meaning that electric power is 30.6 W. 

**Figure 4:**
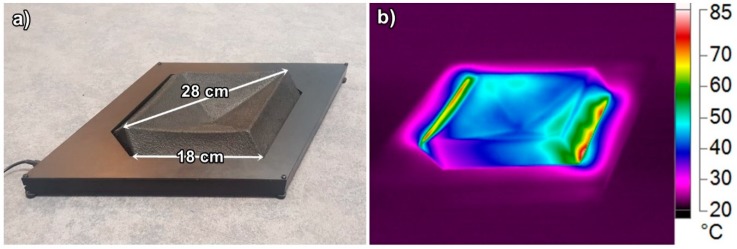
(**a**) Visual image of plywood mounted Salmiakki with dimensions and (**b**) Infrared camera image of heating element prototype Salmiakki at steady state in room temperature.

Finally, the cellulose fiber-based heating element was mounted in a plywood cover that was painted black using wood wax (Osmo Color 3169, Osmo Holz und Color GmbH & Co, Warendorf, Germany). The surface of the Salmiakki heating element was sprayed with varnish to gain glossy surface (Dupli-color Lackspray, Wolvega, Netherland) and electrical connections were installed. Copper tape forming the electrical connection are located on the opposing sides of the prototype Salmiakki and covering 18 cm^2^ area per side. The distance between these copper connectors is 22 cm. As a power source Salmiakki is using 18.5 V, 3.5 A laptop charger (HP, Palo Alto, CA, USA). Infrared camera imaging was performed using a thermal camera (Fluke Ti400, Everett, WA, USA). In Figure 4, it can be observed that the temperature near copper tape conductors is 65–75 °C and a major part of Salmiakki heating element is heated to a comfortable 35–45 °C temperature at steady state in room temperature. Because of rapid heating and cooling properties of CNTs, the heating response of the whole element to steady state is in the order of minutes. 

**Figure 5:**
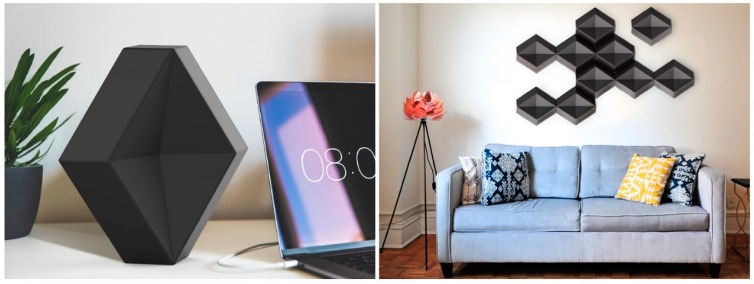
Digital models of Salmiakki heating element.

When calculated, based on the known pricing of raw materials´, the total costs of Salmiakki heating element count less than four euros to manufacture the object (foaming plant investments not accounted for). In addition to the physical prototype, digital models that were rendered as realistic images of the heating element in use were produced to demonstrate how various modular compositions may be applied with various interior settings (see Figure 5). 

The verification of the applicability via collaborative design, materials research, and prototype manufacturing process resulted in the following valid outcomes:

- Better understanding of application properties and overall possibilities of the novel conductive material
- Testing the foam-forming process of the conductive material into a complex three-dimensional object
- Effective dissemination of the research results for high technology readiness level (TRL) 6-7 through exhibiting of the physical prototype and sharing the digitally produced images.
- Deeper perspective of understanding the potential and possibilities of foam forming for producing functional 3D-structures.

## 5. Conclusions

This work focuses on the analysis of conductivity and mechanical strength of NC, CNT, and cellulose pulp based non-woven composite structure. Secondly, application of the foam forming process to prepare conductive non-woven sheets, where nanocellulose acts as a carrier for carbon nanotubes, is studied. Finally, the TRL 6-7 verification and validation programme is reported to establish industrial potential. The results show that it is possible to improve the surfactant selection, the sonication process of the NC-CNT dispersion, and foam forming to achieve the highly even coverage of CNT over the NC network, resulting in very high conductivity of 7.7 S/m. The effect of surfactant removal by acetone washing was studied, but the effects were not significant on the measured mechanical strength and conductivity values. Reason for this might be that the majority of surfactant is removed already during the vacuum assisted moulding process. The applicability and a design product case ‘Salmiakki’ were studied and the advantages of the material system were validated for a heating element application. The product case showed that it is possible to manufacture designed 3D heating element using a minimum amount of materials, processing steps, and hazardous chemicals.

These results mean that it is possible to manufacture conductive non-woven and utilize our innovation using only two processing steps: sonication of the NC-CNT dispersion and foam forming of the 3D structure. The whole process can be done using a minimun amount of materials and hazardous chemicals.

## Figures and Tables

**Table 1:** Material concentrations in different processing steps.

Material	Dispersion	Foam Forming
NC	0.15%	0.1%
CNT	0.3%	0.2%
Surfactant Triton X-100	0.4%	0.25%
Pulp	-	0.35%
Total volume	1800 mL	5500 mL
